# A large, international study on post-transplant glomerular diseases: the TANGO project

**DOI:** 10.1186/s12882-018-1025-z

**Published:** 2018-09-12

**Authors:** Audrey Uffing, Maria José Pérez-Sáez, Gaetano La Manna, Giorgia Comai, Clara Fischman, Samira Farouk, Roberto Ceratti Manfro, Andrea Carla Bauer, Bruno Lichtenfels, Juliana B. Mansur, Hélio Tedesco-Silva, Gianna M. Kirsztajn, Anna Manonelles, Oriol Bestard, Miguel Carlos Riella, Silvia Regina Hokazono, Carlos Arias-Cabrales, Elias David-Neto, Carlucci Gualberto Ventura, Enver Akalin, Omar Mohammed, Eliyahu V. Khankin, Kassem Safa, Paolo Malvezzi, Michelle Marie O’Shaughnessy, Xingxing S. Cheng, Paolo Cravedi, Leonardo V. Riella

**Affiliations:** 1 Renal Division, Brigham & Women’s Hospital, Harvard Medical School, 221 Longwood Ave, Boston, MA 02115 USA; 2 Servicio de Nefrología, Hospital del Mar, Barcelona, Spain; 3 Department of Experimental Diagnostic and Specialty Medicine (DIMES), Nephrology, Dialysis and Renal Transplant Unit, St. Orsola Hospital, University of Bologna, Bologna, Italy; 4 Renal Division, Department of Medicine, Icahn School of Medicine at Mount Sinai, 1 Levy Place, New York, NY 10029 USA; 5 Renal Division, Hospital de Clínicas de Porto Alegre, Porto Alegre, Rio Grande do Sul Brazil; 6 Renal Division, Hospital do Rim, Universidade Federal de Sao Paulo, Sao Paulo, Brazil; 7 Renal Division, Bellvitge University Hospital, Barcelona, Spain; 8 Pro-Renal Foundation/Cajuru University Hospital, Curitiba, Paraná, Brazil; 9 Hospital das Clínicas, University of São Paulo School of Medicine, São Paulo, Brazil; 10 Montefiore Einstein Center for Transplantation, Montefiore Medical Center, Albert Einstein College of Medicine, Bronx, NY USA; 11 Transplant Institute, Beth Israel Deaconess Medical Center, Harvard Medical School, Boston, MA USA; 12 Transplant Center and Division of Nephrology, Massachusetts General Hospital, Harvard Medical School, Boston, MA USA; 13 Service de Néphrologie Dialyse, Aphérèses et Transplantation, Grenoble University Hospital, Grenoble, France; 14 Division of Nephrology, Department of Medicine, Stanford University School of Medicine, Palo Alto, CA USA

**Keywords:** Glomerulonephritis, Registry, Database, Recurrence, Kidney transplant

## Abstract

**Background:**

Long-term outcomes in kidney transplantation (KT) have not significantly improved during the past twenty years. Despite being a leading cause of graft failure, glomerular disease (GD) recurrence remains poorly understood, due to heterogeneity in disease pathogenesis and clinical presentation, reliance on histopathology to confirm disease recurrence, and the low incidence of individual GD subtypes. Large, international cohorts of patients with GD are urgently needed to better understand the disease pathophysiology, predictors of recurrence, and response to therapy.

**Methods:**

The Post-TrANsplant GlOmerular Disease (TANGO) study is an observational, multicenter cohort study initiated in January 2017 that aims to: 1) characterize the natural history of GD after KT, 2) create a biorepository of saliva, blood, urine, stools and kidney tissue samples, and 3) establish a network of patients and centers to support novel therapeutic trials. The study includes 15 centers in America and Europe. Enrollment is open to patients with biopsy-proven GD prior to transplantation, including IgA nephropathy, membranous nephropathy, focal and segmental glomerulosclerosis, atypical hemolytic uremic syndrome, dense-deposit disease, C3 glomerulopathy, complement- and IgG-positive membranoproliferative glomerulonephritis or membranoproliferative glomerulonephritis type I-III (old classification). During phase 1, patient data will be collected in an online database. The biorepository (phase 2) will involve collection of samples from patients for identification of predictors of recurrence, biomarkers of disease activity or response to therapy, and novel pathogenic mechanisms. Finally, through phase 3, we will use our multicenter network of patients and centers to launch interventional studies.

**Discussion:**

Most prior studies of post-transplant GD recurrence are single-center and retrospective, or rely upon registry data that frequently misclassify the cause of kidney disease. Systematically determining GD recurrence rates and predictors of clinical outcomes is essential to improving post-transplant outcomes. Furthermore, accurate molecular phenotyping and biomarker development will allow better understanding of individual GD pathogenesis, and potentially identify novel drug targets for GD in both native and transplanted kidneys. The TANGO study has the potential to tackle GD recurrence through a multicenter design and a comprehensive biorepository.

## Background

Kidney transplantation is the ideal treatment for most patients with chronic kidney failure, providing longer survival and a better quality of life when comparing to dialysis [1, 2]. Unfortunately, improvements in the short-term outcomes have not been paralleled by similar advancements in long-term outcomes [3], with a kidney graft half-life of only around 10 years [4]. Understanding the pathophysiology of chronic allograft injury is essential for providing timely therapeutic interventions and improving allograft survival [5].

Recurrent glomerular disease (GD) after transplant has been described as the third leading cause of graft loss [6]. While some forms of primary kidney disease are associated with higher risk of recurrence and subsequent early graft loss, others are associated with a delayed presentation and slowly progressive course [7]. With the exception of specific mutations for atypical hemolytic uremic syndrome (aHUS) [8] and focal segmental glomerulosclerosis (FSGS) [9], and anti-PLA_2_R [10] and anti-thrombospondin type 1 [11] autoantibodies for membranous nephropathy (MN), no clear genetic, epigenetic or environmental risk factors have been identified to predict the risk of recurrence [12]. Furthermore, observational studies have failed to demonstrate a clear association between GD recurrence and immunosuppression regimens [13].

In the setting of rare diseases, patient registries represent precious tools to characterize the natural history of a condition, to evaluate clinical therapies, to monitor drug safety and to measure quality of care [14]. Given the low incidence and the heterogeneity of post-transplant GD, registries that sample large numbers of patients are required for the collection of sufficient data to facilitate clinical outcomes research. Data quality is another essential ingredient for such registries. The United States Renal Data System (USRDS), a commonly utilized database for epidemiologic research of end-stage renal disease patients, has a large degree of missing data, lacks kidney biopsy data, and frequently misclassifies patients with a diagnosis of GD, preventing robust epidemiologic analyses of GD recurrence [15]. Therefore, ad hoc registries are needed to define the natural history and response to therapy of GD recurrence. Other important elements to consider include the representability of the data, making international registries including patients with different genetic backgrounds and heterogeneous treatments the ideal tool. An international cohort study would also provide the ideal platform upon which to build a large-scale repository of biosamples, which in turn could be used to identify and study biomarkers related to the evolution of the disease and the response to treatment.

We established The Post-Transplant Glomerular Disease (TANGO) study, a large international network of centers to study GD recurrence after renal transplantation (www.tangoxstudy.com) in January 2017. The TANGO study is a multi-phase collaborative project involving retrospective and prospective data collection and biobanking samples to better characterize GD post-transplant.

Herein, we describe the purpose, specific aims and methodology of the TANGO study, setting the foundation for the creation of a shared international biorepository of samples from GD patients and a research network that will facilitate future clinical trials.

### Overarching goals of the TANGO study

1. To define the epidemiology, risk factors, natural history and response to therapy of GD post-transplant (Phase 1).
2. To create a large biorepository of human samples (saliva, blood, peripheral blood mononuclear cells (PBMC), urine, stool, and kidney tissue samples) for biomarker validation and discovery (Phase 2).
3. To develop a network of centers to optimize recruitment and collaboration in clinical trials (Phase 3).

### Specific goals of the TANGO study

1. Phase 1 - Registry:
  - Assess the incidence of GD recurrence after transplant in different countries
  - Describe natural history of post-transplant GD recurrence.
  - Identify risk factors for GD recurrence.
  - Analyze the impact of antirejection therapy on the incidence and severity of GD recurrence.
  - Compare the safety/efficacy profile of different ad hoc therapies used to treat GD recurrence.
2. Phase 2 - Biorepository
  - Establish a standardized protocol for biobanking serial samples post-transplant
  - Identify gene variants or mutations associated with GD recurrence or prognosis.
  - Study gene expression signatures in PBMCs or in kidney tissue cells that predictGD recurrence.
  - Search for serum/urinary predictors of risk of recurrence, activity or response to treatment.
  - Investigate the microbiota and its correlation with disease recurrence.
  - Identify molecular signatures in kidney biopsies related to disease recurrence.
3. Phase 3 - Network
  - Facilitate the recruitment of carefully phenotyped cohorts of patients with primary GD, with or without recurrence in the transplanted graft, for enrolment in academic or industry-sponsored multicenter studies examining pathogenic mechanisms, biomarkers, and therapies for the prevention and treatment of GD recurrence.

## Methods/Design

**Fig. 1:**
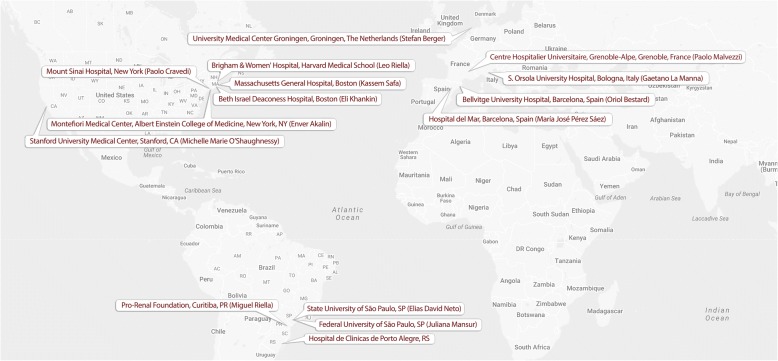
Participating centers in the TANGO Study (image adapted from Google Maps, 2017)

The TANGO study was initiated in January 2017 with the initial number of 15 participating centers in Europe, North-, and South-America (Fig. 1). The study will be composed of three phases:

### Phase 1 – Data registry

Collection of data from patients with or without recurrent GD will be executed by medically trained researchers at each site. A dedicated researcher at the Brigham and Women’s hospital will oversee the quality and completeness of entered data remotely. Scientific oversight, governance and data coordination are provided by the principal investigators (PIs) of the project at each site. Each PI will also be involved in providing feedback on publication goals, logistics and drafted manuscripts from the TANGO Registry. Proposals from participating centers to obtain access to the full dataset in order to study specific glomerular diseases post-transplant will be reviewed by the steering committee, which will initially consist of Leonardo V Riella, Paolo Cravedi, Audrey Uffing and transplant patient representative.

Phase 1 of the TANGO study protocol was submitted and approved by the ethical committee of the Partners Human Research Committee (PHRC) at the Brigham and Women’s hospital in Boston, and at each participating center. In one participating center, the University Medical Center Groningen, ethical approval for phase one was waived by the Medical Ethics review Board (METc UMCG). All protocols are in accordance with International Conference on Harmonization Good Clinical Practice Guidelines and the Declaration of Helsinki. Centers that have an interest of participating in the TANGO-study can send a request to contact@tangoxstudy.com.

#### Inclusion and exclusion criteria

The TANGO study will enroll adult (≥18 years) patients with a biopsy-proven primary GD as the designated cause of their end-stage kidney disease who underwent a kidney transplant starting from January 2005. The glomerular diseases that TANGO Registry includes are listed in Table 1. Complete inclusion/exclusion criteria are presented in Table 2. All clinical data regarding disease history prior to and after kidney transplantation will be manually extracted from the patient’s medical records.

**Table 1:** List of biopsy-proven primary glomerular diseases that are used to identify patients eligible for registration in the TANGO Study

IgA nephropathy Membranous glomerulonephritis Focal and segmental glomerulosclerosis Atypical hemolytic uremic syndrome Membranoproliferative glomerulonephritis old classification type I-III Complement- or IgG-positive membranoproliferative glomerulonephritis Dense-deposit disease C3 glomerulonephritis

**Table 2:** Inclusion/exclusion criteria for registering in TANGO study database

Inclusion criteria	Exclusion criteria
1. ≥18 years-old 2. Biopsy proven GD as underlying cause of end-stage kidney disease (listed in Table 2) 3. Recipient of a kidney transplant after 2005, currently functioning or not 4. With/without recurrence of GD	1. Patients without diagnostic native kidney biopsy 2. Patients with a secondary cause of GD 3. Unable to provide written consent^a^

^a^Not applicable for phase 1

In a few centers, pre-transplant biopsy may not be available for a sizable amount of patients. Subjects with a clinical history strongly suggestive of GD (e.g., sudden onset of nephrotic syndrome, microhematuria, response to steroid therapy etc.), will be included in the study due to the high risk of disease recurrence after transplant. However, their biological samples will be used only in case of biopsy proven GD after transplant. Their clinical data will be collected, but these subjects will not be used for primary epidemiological analyses.

#### Variables and follow-up

At enrollment (time of transplant) and every year thereafter the following data will be collected: patient demographics, renal and other medical past history, kidney transplantation features, history of rejections and glomerular disease occurrence/recurrence post-transplant. Table 3 summarizes the data that will be entered in the online database.

**Table 3:** Study variables required for the TANGO data registry

Variables	
Patient demographics	Subject ID, year of birth, gender, race, height, weight
Kidney history	Cause of kidney disease, dialysis duration, residual urine output, nephrotic proteinuria pre-transplant, native kidney nephrectomy, family history of glomerulonephritis
Other past medical history	Hypertension, diabetes mellitus, coronary artery disease, hepatitis, cancer, autoimmune disease, others
Recipient-Donor Transplant details	Date of transplant, preemptive transplant, prior transplant, donor’s characteristics, cold ischemia time, recipient’s panel reactive antibody, HLA mismatch, donor specific antibody prior transplant, crossmatch, delayed graft function, EBV serology, CMV serology, induction and maintenance immunosuppression drugs
Post-transplant visits (yearly)	Medications, Physical examination, relevant laboratory test post-transplant (including blood and urine), rejection episodes and treatment received, donor specific antibodies development, infections (virus), cancer, other complications, recurrent glomerular disease and treatment
Patient outcome	Graft failure and cause, patient death and cause

#### Database

The TANGO dataset is made available by REDCap^™^(Research Electronic Data Capture), a browser- based, metadata-driven electronic data capture software solution, for designing clinical and translational research databases (https://projectredcap.org). It is widely used in the academic research community: the REDCap^™^ Consortium is a collaborative, international network of more than 2000 institutional partners in over 100 countries, with more than 400,000 total end-users employing the software for more than 200,000 ongoing research studies [16]. Investigators have access to the secure website for entering and accessing patient data online, which will be stored at a secure and confidential location. Individual centers have access to their own recorded data that they can use for analysis, but will not be able to review other center’s data. The study main PIs (L Riella, BWH, Boston and P Cravedi, Mount Sinai, New York) and research coordinators of the project will have access to all records from all centers, except for patient identifiers, which are restricted to the specific center to ensure participant confidentiality.

#### Data analysis, expected sample size and statistical approach

All recorded data will be checked for consistency, errors and missing data to ensure high-quality data. Data from different centers will be combined and used for epidemiological studies, with the main objective to determine incidence of recurrent GD and to assess clinical predictors. We will also analyze response to different therapies, and evaluate other complications such as rejection and infections.

Previous studies showed that recurrence of the glomerular diseases included in the TANGO-study range from 10 to 90% [17], depending, among other variables, on the different diseases, diagnosis criteria, immunosuppressive therapies, and geographic location. Sample size for population proportion is calculated per disease and is based on the primary outcome for phase 1: the proportion of recurrence of glomerular disease post-transplant. Estimated sample sizes are shown in Table 4 and are calculated using the most conservative proportion of recurrence (i.e., closest to 0.5) from previously reported ranges [18, 19], a CI of 90% and a margin of error of 5%. Consequently, each of the 15 participating centers has to include 15–18 patients per disease. For IgA-nephropathy, MN, FSGS and MPGN this seems a reasonable number to achieve over an inclusion period of 10 years. aHUS, however, has an incidence of 1–2 cases per million, mainly occurring in childhood [19]. Hence, in an adult transplantation population, our study is unlikely to achieve a sample size of 270 for aHUS and precision of the estimated rate of recurrence will be reduced.

**Table 4:** Sample size calculations per disease entity

Disease	Estimated sample size
IgA-nephropathy	227
Membranous nephropathy	260
Focal segmental glomerulosclerosis	260
Membranoproliferative glomerulonephritis	270
Atypical hemolytic uremic syndrome	270
Total	1287

Data will be analyzed in a de-identified fashion using Stata software (StataIC-15, StataCorp LLC). For categorical data Fishers Exact test or Pearsons’ chi-square tests will be used. Continuous data will be plotted and tested by the Shapiro-Wilk test to confirm normal distribution. Normally distributed data will be analyzed by t-test. For non-parametric data, Mann-Whitney U test will be used. Cox-proportional Hazards will determine hazard ratios and will be tested using Martingale Residuals. Tests will be 2-sided and *p*-values < 0.05 will be considered as statistically significant.

### Phase 2 – Biorepository

A large repository of biological samples will be instrumental to better understand GD pathogenesis and to identify biomarkers of disease recurrence.

#### Sample collection

Biobanking of samples requires an established infrastructure at the participating center. Some of the participating centers already biobank samples and we will link samples available locally to our global database. At the Brigham and Women’s hospital in Boston, a biobanking protocol was submitted and approved by the PHRC (protocol number 2017P000298). Ethical approval of sample collection at other centers is currently ongoing.

For participating TANGO centers, relevant samples from saliva, whole blood, serum, urine and stool will be stored at the time of transplantation and at the time of GD recurrence. If GD recurs, leftover kidney tissue samples from biopsies will be stored at that time. For patients already transplanted with a diagnosis of primary GD, samples will be obtained post-transplant with or without recurrence, at yearly intervals and at the time of each graft biopsy. Funds for the creation of broader international biorepository will be pursued during 2018 in order to allow the generation of an ad hoc biobank where saliva, blood, stool and urine samples will be collected before transplant, at 6 months, and every year thereafter.

Aliquots of urine supernatants, urine-cell pellets and serum samples, genomic DNA, blood Pax gene tubes for RNA analysis, and biopsy samples will be stored and banked in a − 80 °C temperature freezer. PBMC will be kept in liquid nitrogen.

#### Sample processing and analysis

##### Saliva

Saliva will be obtained in DNA saliva collection tubes (Oragene, DNA genotek) and will be used for DNA-extraction and genotyping, to identify gene variants or mutations associated with the recurrence or the prognosis of the GD. DNA-analysis will also be valuable to categorize patients more accurately in biomarker studies, where samples from patients with a genetic cause of GD can be treated as a separate entity.

##### Whole blood

Whole blood will be stored at − 80 °C in blood RNA tubes, for extraction of RNA. RNA quality will be assessed using Bioanalyzer and will be used to build RNAseq libraries and sequenced on a HiSeq 2500. The results will be used to identify gene expression signatures predictive of GN recurrence.

##### PBMC

PBMC will be isolated from whole blood by Ficoll separation within 6 h of collection, and frozen using a standard operating procedure [20]. Cell will be used for flow cytometry, mass cytometry (CyTOF), and single-cell RNA sequence analyses.

##### Serum and urine

Aliquots of serum will be frozen at − 80 °C. Urine samples will be centrifuged at 3200 rpm for 5 min at 4 °C within 4 h of collection. The sediment will be washed and stored at − 80 °C in RNA later for gene expression studies. Supernatants will be divided into aliquots and stored at − 80 °C.

Several proteomic and metabolomic assays can be performed with serum and urine samples, for evaluation of potential novel biomarkers to pathogenic pathways in recurrent GD or response to pharmacologic intervention. Besides well-established immunoassays such as ELISA and Luminex, urine and serum can be analyzed by new high-output proteomics such as the SOMAscan. The SOMAscan is a high multiplex, high sensitivity aptamer-based immune like protein and biomarker discovery platform, that can simultaneously quantify over 1300 proteins [21]. The high number of proteins being analyzed provides the opportunity to discover pathways that involve multiple proteins, and eventually lead to GD. SOMAscan data is validated by ELISA. In subsequent analysis, actual pathogenicity of proteins of interest can be assessed in mice-models [22], kidney-organoid models [23], or in specific disease models that assess human-specific glomerular injury [24].

##### Kidney biopsy tissue

For patients who have undergone a kidney biopsy based on clinical or protocol indication and have enrolled in this study, excess biopsy material (if available) will be requested and stored to perform further analyses of immune biomarkers and correlate with findings from biomarkers on blood and urine.

##### Stool

Stool samples will be obtained using stool collection devices (Ability Building Center, inc) and stored in RNA later in − 80 degrees Celsius. The importance of the microbiota has expanded in recent years, including evidence that it may influence transplant outcomes [25]. With stool samples, we will be able to determine the composition of the gut microbiota and correlate with GD recurrence post-transplant. This may be particularly important for certain GD such as IgA nephropathy in which this subtype of immunoglobulin is predominantly secreted on intestinal mucosa as a response to intraluminal pathogens. Specifically, we will extract DNA from stools and perform 16S and 18S rRNA gene sequencing and total DNA sequencing for shotgun metagenomics.

Since the variety of collected samples require diverse analysis methods, samples will be analyzed in different labs that have expertise on the particular assay. For each individual study, however, samples will be analyzed at the same institute.

Ideas for collaboration or requests from non-participating centers to use data and/or samples are appreciated and will be reviewed by the steering committee. Priority in usage of data and samples will always be given to participating centers.

### Phase 3 - clinical trial network

Phase 1 and 2 will provide the specific setting for the development of a large dataset of patients eligible for interventional studies. Currently available and newly identified biomarkers will enable the identification of patients at highest risk for recurrence or renal disease progression, which would help in increasing the statistical power of the clinical studies. With the data obtained from the preliminary phases using the samples from the biobank, interventional studies can be designed and conducted with grants from public institutions, foundations or pharmaceutical companies.

## Discussion

Post-transplant GD recurrence represents the third leading cause of long-term graft loss [6]. Despite its relevance, the incidence, the natural history and the risk factors for GD recurrence remain poorly understood. Several factors may contribute to this lack of knowledge, including the heterogeneity of the different diseases and the absence of large registries that accurately describe the evolution of patients with GD after transplant. Most registries of GD recurrence are single center. On the other hand, larger renal registry data frequently misclassify the cause of kidney disease, confounding estimates of GD recurrence after transplant [15]. As an example, the reported incidence of focal segmental glomerulosclerosis (FSGS) recurrence after transplant may varies from 10 to more than 50% in recent studies [26–28]. This probably relates to diverse inclusion criteria, population characteristics, underlying disease mechanisms, management policies (e.g., including surveillance biopsies or not) and follow-up times across studies. Similar limitations can be found in terms of prognosis when the registries have not been designed ad hoc [29–31]. Besides the description of the natural history of the GD recurrence, the identification of risk factors for GD recurrence or biomarkers of disease activity would reliably inform patient care [13, 32, 33]. To this end, systematic and comprehensive data collection from larger numbers of patients followed-up for longer time would greatly enhance understanding of disease epidemiology and potentially improve patient outcomes. In these terms, the TANGO study is an initiative that aims to respond to these unmet needs: i.e. a large-scale, systematically collected, multi-center registry of patients with biopsy proven (gold standard diagnostic test) GD prior to KT. The TANGO study is a detailed international multicenter registry designed to describe the natural history of GD after transplant as well as the identification of potential risk factors for GD recurrence. To the best of our knowledge, this is the first international multicenter cohort study to examine GD recurrence and evolution after transplantation. Creation of a large biorepository will also facilitate mechanistic studies investigating disease pathogenesis and the identification of noninvasive, reliable immune monitoring assays to predict disease recurrence and response to treatment. Similarly designed large international registries have been postulated to collect high quality data about other rare diseases such as atypical hemolytic uremic syndrome or glomerular diseases in native kidneys [34, 35].

### Strengths and limitations

The strengths of the study will include: the proposed large size of the cohort, the requirement for a biopsy proven diagnosis of GD, the systematic collection of detailed, long-term, clinical data; and the diversity of the included participants from different geographic regions and of different racial/ethnic backgrounds. Limitations include: a lack of surveillance biopsies in some participating centers, which may limit our capacity to identify recurrence in the absence of clinical abnormalities, and the absence of centralized pathology interpretation of biopsies, which will also represent a bias of the present study. Nonetheless, we expect this study to be instrumental in elucidating risk factors, pathogenesis, and therapeutic targets for post-transplant GD.

## Abbreviations

FSGS: Focal Segmental Glomerulosclerosis
GD: Glomerular disease
KT: Kidney transplantation
PBMC: Peripheral blood mononuclear cells
PI: Principal investigator
TANGO: Post-TrANsplant GlOmerular Disease
USRDS: United States Renal Data System
